# Electromyographic Study on the Inhibitory Effects of Local Cold- and Hot-Water Bathing of the Upper Limb on Finger Flexor α-Motor Neuron Activity

**DOI:** 10.7759/cureus.84954

**Published:** 2025-05-28

**Authors:** Mayu Komatsu, Masaaki Nakajima

**Affiliations:** 1 Graduate School of Health Sciences, Kibi International University, Takahashi, JPN

**Keywords:** cold stimulation, muscle tone suppression, pain, spasticity treatment, α-motor neuron activity inhibition

## Abstract

This study evaluated the inhibitory effects of cold and heat stimulation on α-ｍotor neuron activity and the associated pain, exploring their clinical applications.

Twenty-four healthy young adults participated, undergoing four conditions: (1) cold-water finger immersion, (2) warm-water finger immersion, (3) warm-water forearm immersion, and (4) a control condition. Assessments included grip strength, integrated electromyography (IEMG) of finger flexor muscles, skin temperature, and pain perception.

Cold-water finger immersion involved three sets of 5-second ice water immersion with 2-second breaks. Warm-water immersion (finger and forearm) lasted 10 min at 42°C. Measurements were taken before and at 5-minute intervals up to 20 min post-intervention, with pain assessed via the Numerical Rating Scale (NRS).

Cold-water finger immersion significantly reduced grip strength, IEMG, and skin temperature while increasing NRS scores. In contrast, warm-water immersion had no significant effect. The cold-water condition also showed a prolonged skin temperature drop. These findings confirm that cold stimulation inhibits α-motor neuron activity, primarily due to pain, though the effect is temporary.

Cold stimulation may improve range-of-motion (ROM) exercise performance, potentially preventing joint contractures. This suggests that cryotherapy could be a valuable approach for managing spasticity in post-stroke patients. Since finger flexor spasticity impairs activities of daily living (ADL) and quality of life (QOL), reducing spasticity is crucial for ROM exercises.

## Introduction

Finger flexor spasticity in stroke paraplegia patients

In stroke paraplegia patients, finger flexor spasticity leads to abnormal muscle tone, impairing hand function, causing joint contractures and pain [1], and reducing activities of daily living (ADL) and quality of life (QOL) [2]. Severe spasticity limits the joint range of motion (ROM), increasing contracture risk.

Inhibition of spinal motor neuron activity by thermal and cold stimulation

Cold stimulation with ice water can temporarily reduce upper limb spasticity [3]. Knutsson et al. [4,5] found that spinal cooling quickly decreased muscle tone, likely due to reduced γ efferent nerve activity.

Thermal stimulation also suppresses abnormal muscle tone. Fountain et al. [6] reported that heat increased Golgi tendon organ Ib fiber activity, raising motor neuron thresholds. However, the extent and duration of these effects remain unclear. Cold stimulation may also cause pain, which could affect spinal motor neuron activity [7].

This study analyzed electromyography (EMG) of the deep and superficial finger flexors to evaluate the effects of hot and cold stimuli on finger flexion and spinal motor neuron activity in healthy subjects.

## Materials and methods

Study design

This study was designed as a randomized crossover trial.

Study population and sample size

The participants of this study were university students from Kibi International University, with a total of 24 healthy students (13 males and 11 females) taking part in the study. The mean age of the participants was 20.3 ± 1.0 years, and the mean body mass index (BMI) was 21.2 ± 2.0.

The study was conducted from October to December 2023, and all experiments were carried out at Kibi International University.

Experimental protocol

Subjects performed four different intervention tasks on different days.

To prevent the pain induced by cold-water immersion from affecting subsequent intervention sessions, a washout period of more than three days was implemented.

**Figure 1:**
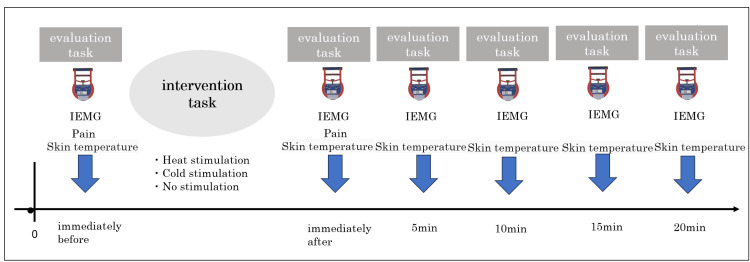
Experimental protocol Each subject underwent four intervention tasks (hot-water hand bathing, hot-water hand and forearm bathing, cold-water hand bathing, and no load) on different days. Intervention tasks were performed immediately before and after the intervention task, and every 5 min until 20 min had elapsed. IEMG, integrated electromyography.

Measurements were taken immediately before, immediately after, and 5, 10, 15, and 20 min after the interventions, including grip strength, skin temperature, and EMG from the flexor digitorum superficialis and flexor digitorum profundus. Pain was assessed immediately after the intervention. The room temperature was adjusted to 25 ± 1℃. The order in which each intervention task was performed was randomized (Figure 1).

However, blinding was difficult to implement in this study because it was necessary to promptly evaluate the effects of skin temperature changes induced by thermal stimulation.

Intervention task 

There were four intervention tasks: cold-water hand bathing, hot-water hand bathing, hot-water hand and forearm bathing, and no load (control). 

*Cold-Water Hand* *Bathing*

**Figure 2:**
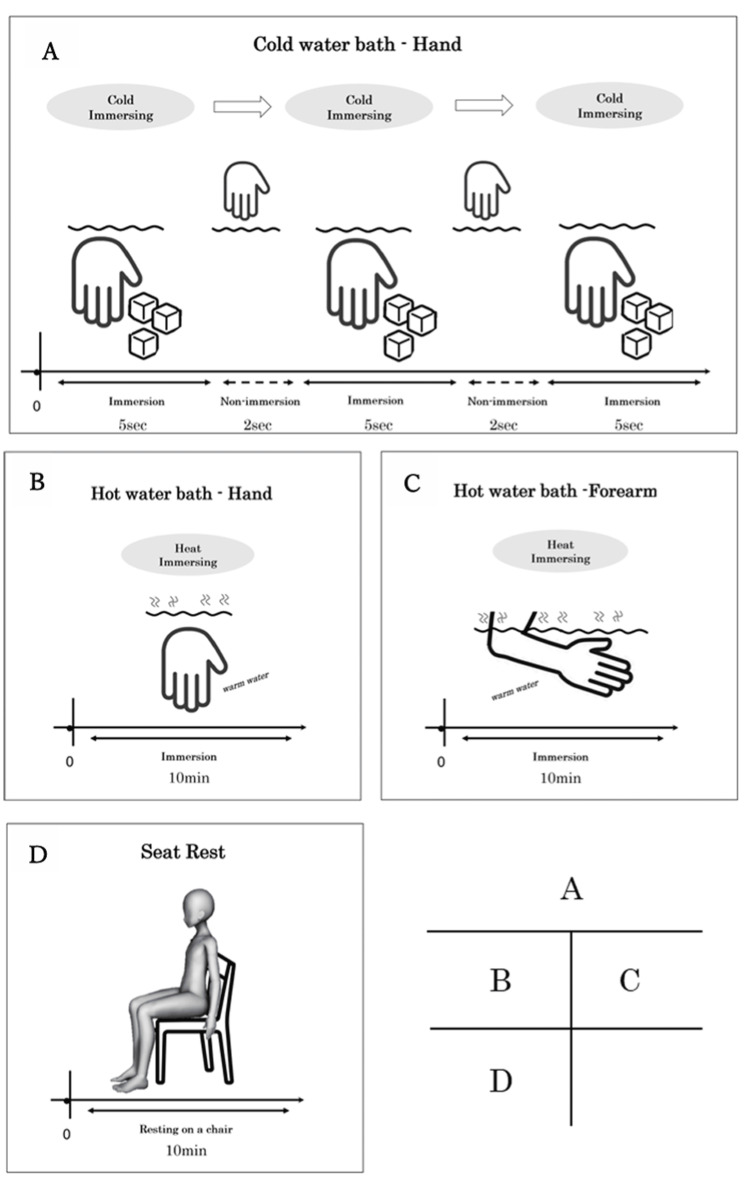
Intervention tasks A. Cold-water hand bathing: The caregiver immersed the subject's hand up to the stromal process and moved it in a slow figure-eight motion; three 5-second immersions with a 2-second break in between were performed. The temperature of the bath water was 0℃. B. Hot-water hand bathing: The subject immersed his hand up to the styloid process in 42℃ warm water and held it there for 10 min. C. Hot-water hand and forearm bathing: The hand and forearm were immersed in warm water at 42°C and held for 10 min. D. No load (control): The no-load condition was also used as a control. The subject spent 10 min in a sitting position without a local bath.

For the cold-water hand bathing, the hand was immersed distal to the styloid process in ice water in a styrofoam container measuring 18.5 cm x 29.0 cm x 22.5 cm (internal dimensions, length, width, and depth). Cold water was provided in containers filled with tap water and ice. The water temperature was 0℃; 5-second immersions were performed three times with a 2-second break in between (Figure 2A) using the method described by Patricia M. Davies [3].

*Hot-Water Hand* *Bathing*

For this intervention, the hand was immersed up to the styloid process in 42℃ water using an upper limb whirlpool bathtub (Hydrobubbler BB-4000; Sakai Medical Co., Ltd., Tokyo, Japan) (Figure 2B). Based on common practice in physical therapy, the immersion duration was set to 10 minutes in this experiment [8,9].

*Hot-Water Hand and Forearm* *Bathing*

For the hot-water hand and forearm bathing, the hand and forearm were immersed in 42℃ water in an upper limb whirlpool bathtub (Hydrobubbler BB-4000; Sakai Medical Co., Ltd., Tokyo, Japan) for 10 min (Figure 2C) [8,9]. 

No Load (Control)

The no-load (control) group consisted of 10 min of sitting at rest without doing anything (Figure 2D). 

Study measures

Evaluation Task Flexor Digitorum Profundus

The evaluation task consisted of measuring the maximum voluntary grip strength and integrated electromyography (IEMG) values of the flexor digitorum superficialis and digitorum profundus muscles during exercise, skin temperature, and pain. The flexor digitorum profundus and flexor digitorum profundus were selected as the primary action muscles for grip strength testing. IEMG values were used for evaluation because they reflect the activity of α-motor neurons [10]. To measure grip strength, the subjects were removed from the bath water, wiped with a bath towel, and promptly held in a grip strength tester for 3 s at maximum voluntary effort. EMG was derived from the flexor digitorum superficialis and digitorum profundus muscles, and the amplified potentials were converted to analog-digital using PowerLab (ADInstruments Pty Ltd, Nagoya, Japan) and stored on a laptop computer using the data analysis software LabChart 8 (ADInstruments Pty Ltd, Nagoya, Japan) [11,12]. The data were stored on a laptop. IEMG values were calculated by integrating the middle of the obtained waveforms for 1 s of stability.

**Figure 3:**
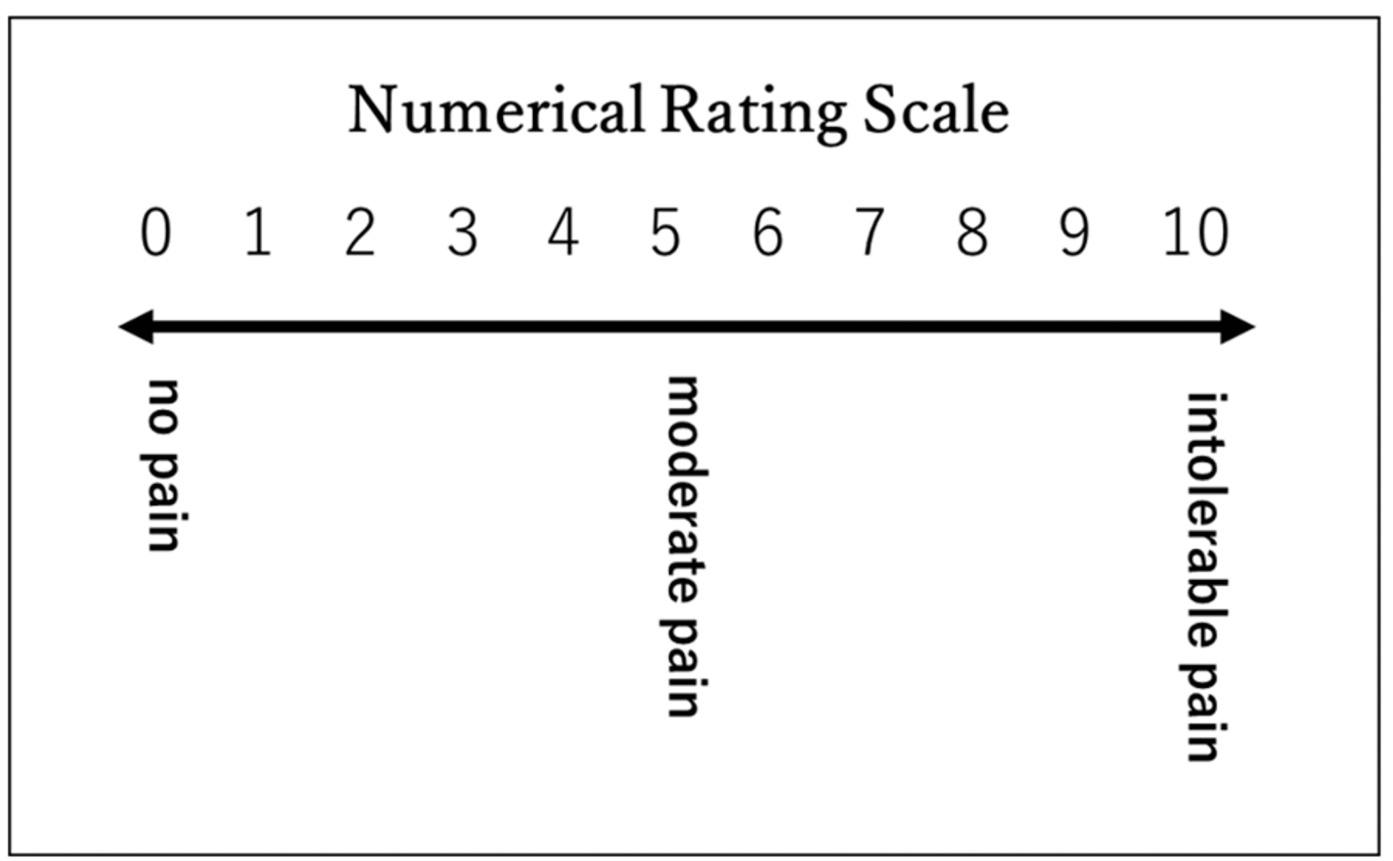
Numerical Rating Scale (NRS) The NRS is a rating scale that asks for a pain score on a scale of 0 to 10, with 0 being no pain at all and 10 being the greatest pain imaginable.

Skin temperature and pain were assessed immediately after the intervention. The skin temperature on the palm was measured using a noncontact thermometer (Spot Thermometer TA-0510; Minolta Camera Co., Ltd., Osaka, Japan). Pain was assessed using a Numerical Rating Scale (NRS) (Figure 3). 

Ethics statement

This study was approved by the Ethical Review Committee of Kibi International University (approval number: 23-28). Before the start of the experiment, participants were provided with both written and oral explanations of the study, ensuring their full understanding before obtaining written informed consent. All participants provided informed consent for their participation in the study. After obtaining consent, each participant was asked to complete a medical history questionnaire. Participants with a history of upper limb-related issues were excluded from the study.

Statistical analysis

All results of this study are presented as means ± standard deviation. A repeated two-way analysis of variance (ANOVA) was performed to evaluate the main effects of time and the interaction between time and local bath conditions on muscle strength, IEMG of the flexor digitorum profundus, and the skin temperature. Multiple comparisons were performed using Tukey’s honestly significant difference (HSD) test when significant differences were found in the two-way ANOVA. The Wilcoxon signed-rank test was used to test the NRS values immediately after cold- and warm-water hand bathing interventions. Statistical analyses were performed using IBM SPSS Statistics version 29 (Armonk, NY: IBM Corp), with a statistical significance level of less than 5%.

## Results

Change in grip strength for each local bath condition 

A two-way ANOVA on grip strength confirmed that both the main effect of time on grip strength (F(5, 88) = 20.658, p < 0.001) and the interaction between the local bath condition and time (F(15, 270) = 6.655, p < 0.001) were significant (Table 1). 

**Table 1:** Change in grip strength values for each local bath condition IEMG, integrated electromyography. *Indicates a statistically significant difference at p < 0.001.

Thermal stimulation	Pre	Post	5 min	10 min	15 min	20 min
No load, Control	31.0 ± 10.6	30.9 ± 10.2	30.6 ± 9.8	30.7 ± 9.4	30.2 ± 9.6	30.9 ± 9.8
Cold-water hand bathing	32.1 ± 10.7	21.6 ± 12.5*	31.2 ± 10.6	30.7 ± 9.6	31.0 ± 9.9	29.8 ± 8.9
Hot-water hand bathing	31.5 ± 9.7	30.6 ± 9.7	30.2 ± 9.9	30.0 ± 9.4	29.6 ± 9.0	29.9 ± 9.5
Hot-water hand and forearm bathing	31.4 ± 9.9	31.3 ± 10.7	31.0 ± 9.7	29.7 ± 9.4	29.1 ± 9.3	29.6 ± 9.5

Results of multiple comparisons using Tukey's HSD showed that the grip strength was lower in the cold-water bathing condition immediately after the intervention (p < 0.05). However, no significant differences were observed after 5 min of the intervention.

Changes in IEMG of the flexor digitorum superficialis and flexor digitorum profundus under each local bath condition 

Statistical processing was performed on the IEMG values for each local bath condition for the flexor digitorum superficialis and digitorum profundus, which are the primary working muscles for grip strength.

Changes in flexor digitorum superficialis IEMG for each local bath condition 

A two-way analysis of the flexor digitorum superficialis IEMG confirmed that the main effect of time (F(5, 88) = 8.882, p < 0.001) and the interaction between condition and time (F(15, 270) = 4.565, p < 0.001) were significant (Table 2).

**Table 2:** Changes in flexor digitorum superficialis IEMG for each local bath condition IEMG, integrated electromyography. *Indicates a statistically significant difference at p < 0.001.

Thermal stimulation	Pre	Post	5 min	10 min	15 min	20 min
No load, Control	1242.3 ± 386.6	1247.8 ± 310.6	1229.0 ± 330.6	1294.3 ± 314.6	1280.4 ± 309.7	1315.2 ± 287.1
Cold-water hand bathing	1277.9 ± 299.0	865.8 ± 388.4*	1256.8 ± 305.7	1232.5 ± 305.0	1268.2 ± 294.4	1266.9 ± 290.3
Hot-water hand bathing	1318.3 ± 268.5	1231.2 ± 267.8	1268.4 ± 328.8	1249.6 ± 296.6	1275.0 ± 281.9	1272.2 ± 324.5
Hot-water hand and forearm bathing	1278.8 ± 299.1	1195.5 ± 304.3	1239.9 ± 290.0	1200.4 ± 288.3	1152.6 ± 352.3	1231.8 ± 285.8

The results of multiple comparisons showed a decrease immediately after the intervention in the cold-water hand bath condition (p < 0.05). However, no significant differences were observed between these values after 5 min of the intervention.

Changes in flexor digitorum profundus IEMG for each local bath condition 

A two-way ANOVA on the flexor digitorum profundus IEMG confirmed that both the main effect of time (F(5, 88) = 12.573, p < 0.001) and the interaction between condition and time (F(15, 270) = 4.358, p < 0.001) were significant (Table 3). 

**Table 3:** Changes in flexor digitorum profundus IEMG in each local bath condition IEMG, integrated electromyography. *Indicates a statistically significant difference at p < 0.001.

Thermal stimulation	Pre	Post	5 min	10 min	15 min	20 min
No load, Control	1520.9 ± 196.6	1488.5 ± 205.5	1482.5 ± 204.7	1512.0 ± 141.2	1498.7 ± 136.5	1515.7 ± 139.7
Cold-water hand bathing	1435.8 ± 219.8	920.4 ± 445.3*	1370.1 ± 213.0	1425.4 ± 189.2	1415.4 ± 211.2	1416.2 ± 260.6
Hot-water hand bathing	1395.6 ± 184.2	1355.4 ± 187.9	1378.0 ± 165.4	1326.7 ± 169.8	1349.9 ± 176.6	1344.2 ± 223.0
Hot-water hand and forearm bathing	1446.7 ± 200.4	1314.2 ± 242.6	1371.1 ± 168.7	1371.6 ± 203.5	1390.2 ± 206.8	1326.7 ± 283.5

The results of multiple comparisons showed that the flexor digitorum profundus IEMG decreased immediately after the intervention in the cold-water hand bath condition (p < 0.05). However, no significant difference was observed between these values after 5 min of the intervention.

Changes in skin temperature for each local bath condition 

A two-way ANOVA of the skin temperature confirmed that both the main effect of time (F(5, 88) = 36.187, p < 0.001) and the interaction between condition and time (F(15, 270) = 7.893, p < 0.001) were significant (Table 4). 

**Table 4:** Changes in skin temperature in each local bath condition *Indicates a statistically significant difference at p < 0.001. **Indicates a statistically significant difference at p < 0.05.

Thermal stimulation	Pre	Post	5 min	10 min	15 min	20 min
No load, Control	31.8 ± 2.3	32.4 ± 2.2	33.1 ± 1.5	33.3 ± 1.5	33.2 ± 1.5	33.3 ± 1.5
Cold-water hand bathing	32.3 ± 2.1	16.5 ± 4.1*	30.9 ± 2.5**	32.2 ± 2.0	32.7 ± 2.1	33.0 ± 1.9
Hot-water hand bathing	32.4 ± 2.1	36.0 ± 0.9	34.5 ± 0.9	34.3 ± 0.7	34.2 ± 0.8	34.0 ± 0.8
Hot-water hand and forearm bathing	32.3 ± 1.8	35.8 ± 4.1	34.3 ± 0.6	34.0 ± 0.7	34.0 ± 0.7	33.7 ± 0.9

Multiple comparisons showed that the skin temperature was lower immediately and after 5 min of intervention in the cold-water hand bath conditions (p < 0.05).

Changes in pain in each local bath condition 

NRS values were significantly higher immediately after the cold-water hand bath intervention (z = -4.197, p < 0.0001) (Table 5).

**Table 5:** NRS during cold-water and hot-water bathings of the hands NRS, Numerical Rating Scale. *Indicates a statistically significant difference at p < 0.0001.

Thermal stimulation	Pre	Post
Cold-water hand bathing	0	7.0 ± 2.0*
Hot-water hand bathing	0	0.9 ± 1.6

## Discussion

Summary of results

In this study, the effects of cold-water hand bathing, hot-water hand bathing, and hot-water hand and forearm bathing on the activity of α-motor neurons in the flexor digitorum superficialis and digitorum profundus were examined in healthy young subjects using grip strength and IEMGs as indices. Pain associated with temperature stimulation was also evaluated. The results showed that cold-water hand bathing reduced the grip strength and IEMGs, while hot-water hand bathing and hot-water hand and forearm bathing did not decrease grip strength and IEMGs. Cold-water hand bathing is suggested to inhibit α-motor neuron activity. Cold-water hand bathing also decreased the skin temperature immediately and 5 min after the intervention. The NRS score, a pain sensory evaluation index, was higher immediately after the cold-water hand bathing intervention than immediately after the hot-water hand bathing intervention.

Description of results

Cold-water hand bathing inhibited the spinal motoneuron activity, whereas hot-water hand bathing and hot-water hand and forearm bathing were not able to inhibit spinal motoneuron activity. Suppression of α-motor neuron activity by temperature and pain stimuli has also been discussed.

Cold and Hot Stimuli to the Skin and Inhibition of α-Motor Neuron Activity

Cold stimuli to the skin send afferent signals to the spinal cord via cold receptors. This signal activates inhibitory interneurons in the spinal cord, particularly Ia inhibitory interneurons, which reduce excitatory inputs to α-motor neurons and inhibit muscle contraction. Cold stimulation of the skin also reduces the activity of γ motor neurons; reduced activity of γ motor neurons reduces the sensitivity of muscle spindles, resulting in reduced input to α-motor neurons [4,5].

Thermal stimulation of the skin increases the firing rate of type Ib fibers from the Golgi tendon organ and raises the threshold of α-motor neurons. In addition, an increase in muscle temperature decreases the firing rate of type II muscle spindle centrifugal fibers and gamma centrifugal fibers, resulting in a decrease in the firing rate of α-motor neurons [10-12].

As shown above, cold and hot stimulation of the skin and hot stimulation of muscles neurologically inhibit the α-motor neuron activity.

Skin Temperature, Pain Stimuli, and Inhibition of α-Motor Neuron Activity

In general, cold stimuli cause pain at temperatures below 15°C [13]. Cold stimulation activates transient receptor potential ankyrin 1 (TRPA1) and transient receptor potential cation channel subfamily M member 8 (TRPM8) receptor ion channels, which induce pain receptor ion channels, leading to pain [14]. Thermal stimulation causes pain at temperatures above 43°C [15]. Heat stimulation activates transient receptor potential vanilloid 1 (TRPV1) ion channels, leading to pain [15].

Pain and Maximal Voluntary Muscle Inhibition

Pain affects maximal voluntary muscle strength [16-18] and causes muscle weakness through inhibitory neural mechanisms. Pain activates inhibitory nerve reflexes in the spinal cord and cortex, thereby reducing the output of muscle activity. This inhibits muscle contraction during painful movements.

Sauro Salomoni et al. [18] reported that in the setting of acute experimental knee pain, pain reduced the maximal voluntary isometric knee extension force by 9.3%. Pain-induced reduction in maximal voluntary muscle strength is primarily due to a deficit in the maximal voluntary drive (maximal voluntary drive).

Serajul I Khan et al. [19] reported that experimental muscle soreness reduced the maximal voluntary contraction torque of the elbow flexors by approximately 5%. However, no significant effect was noted on the voluntary activation levels as assessed using motor point stimulation and transcranial magnetic stimulation.

In the previous studies of Knutsson E [5], it has been reported that the spasticity-reducing effects of cold stimulation using an ice pack on spastic muscles in patients with spasticity persist for approximately 30 min to 1 h. This suggests that the physiological inhibitory effects on α-motor neurons last for at least 30 min. However, in the present study, the inhibitory effect of cold-water hand bathing on α-motor neurons did not persist for even 5 min. Furthermore, the previous study reported that no pain or discomfort occurred after the intervention.

This implies that the α-motor neuron inhibition observed in the present study following cold-water hand bathing was not due to physiological effects but rather due to the occurrence of pain. Supporting this interpretation, the NRS pain score immediately after cold-water hand bathing was high (7.0 ± 2.0), along with a decrease in grip strength and IEMG. Although skin temperature remained decreased even 5 min after the intervention, the skin temperature at 5 min post-intervention was 30.9 ± 2.5°C, which was unlikely to induce pain. Therefore, α-motor neuron inhibition was not observed at this time point.

Limitations of the study

In this study, we evaluated the inhibitory effects of cold-water finger immersion and warm-water local upper limb immersion on α-motoneuron activity in healthy young individuals without spasticity. Therefore, it is difficult to directly apply our findings to patients with spasticity following stroke. Future research should investigate the inhibitory effects of cold-water finger immersion and warm-water local upper limb immersion on α-motoneuron activity in actual stroke patients with spasticity.

## Conclusions

This study demonstrated that cold-water immersion of the fingers suppresses α-motoneuron activity, leading to a decrease in grip strength and IEMG. Furthermore, our findings suggest that the sensation of pain associated with cold stimulation contributes to this inhibitory effect. In particular, we identified that the reduction in skin temperature caused by cold-water immersion, along with the strong pain sensation it induces, serves as a primary factor in triggering α-motoneuron inhibition.

These findings indicate that pain stimulation may function as an effective trigger for reducing abnormal muscle tone caused by increased muscle tension. This expands the potential applications of cryotherapy in the treatment of spasticity. Clinically, the transient suppression of α-motoneuron activity induced by cold-water immersion of the fingers may help alleviate abnormal muscle tone in stroke patients with hemiparesis, thereby facilitating smooth execution of ROM exercises. However, given that cold-water immersion can cause significant pain, careful consideration should be given to minimizing patient discomfort, along with providing appropriate explanations and precautions.

Based on these findings, future research is expected to contribute to the development of new therapeutic strategies for managing spasticity in stroke patients.

## Figures and Tables

**Figure 4:**
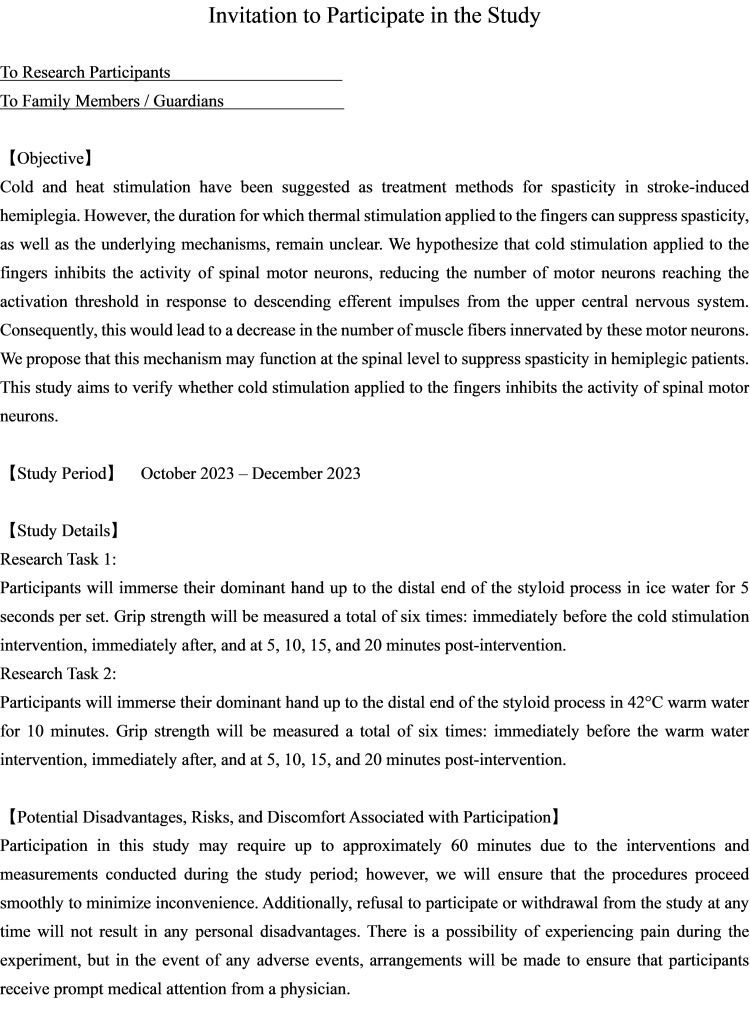
Information sheet for participation in the experiment

**Figure 5:**
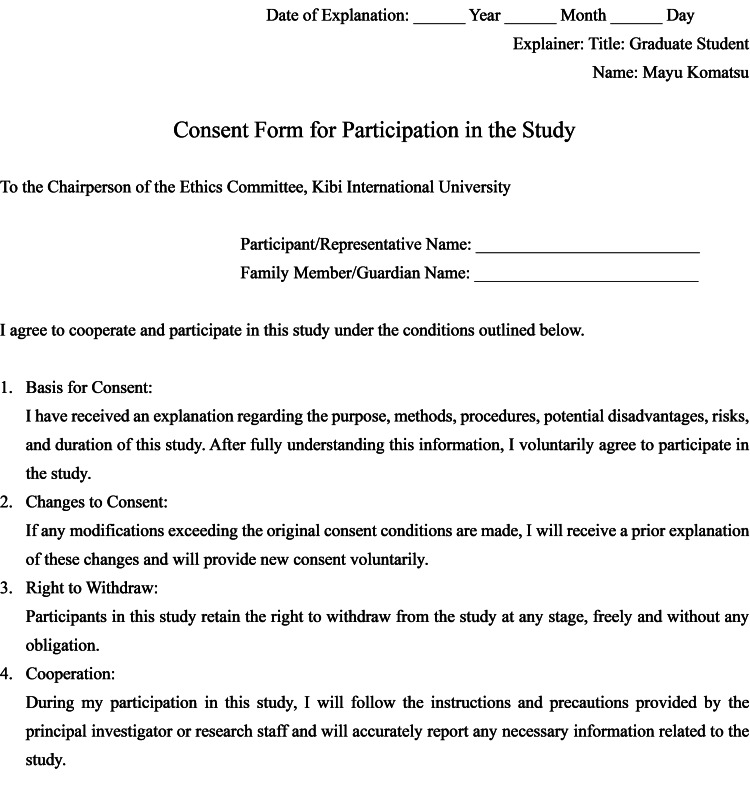
Consent form for the experiment
